# Terahertz Irradiation Improves Cognitive Impairments and Attenuates Alzheimer’s Neuropathology in the APP^SWE^/PS1^DE9^ Mouse: A Novel Therapeutic Intervention for Alzheimer’s Disease

**DOI:** 10.1007/s12264-023-01145-3

**Published:** 2023-11-16

**Authors:** Jun Zhang, Yixin Chen, Yarui Zhao, Panpan Wang, Hongbin Ding, Cong Liu, Junhong Lyu, Weidong Le

**Affiliations:** 1 Liaoning Provincial Key Laboratory for Research on the Pathogenic Mechanisms of Neurological Diseases, the First Affiliated Hospital, Dalian Medical University, Dalian, 116011 China; 2 School of Physics, Dalian University of Technology, Dalian, 116024 China; 3 Interdisciplinary Research Center on Biology and Chemistry, Shanghai Institute of Organic Chemistry, Chinese Academy of Sciences, Shanghai, 201210 China; 4 Advanced Research Institute, Chinese Academy of Sciences, Shanghai, 201203 China; 5 Department of Neurology and Institute of Neurology, Sichuan Academy of Medical Sciences-Sichuan Provincial Hospital, Medical School, University of Electronic Science and Technology of China, Chengdu, 610072 China

**Keywords:** Alzheimer’s disease, Cognitive impairment, Senile plaque, Tau protein, Terahertz

## Abstract

Alzheimer's disease (AD) is a progressive neurodegenerative disease characterized by the deposition of amyloid-β (Aβ), neurofibrillary tangles, neuroinflammation, and neurodegeneration in the brain. In recent years, considering the unsatisfied benefits of pharmacological therapies, non-pharmacological therapy has become a research hotspot for AD intervention. Terahertz (THz) waves with a range between microwave and infrared regions in the electromagnetic spectrum and high permeability to a wide range of materials have great potential in the bioengineering field. However, its biological impacts on the central nervous system, under either physiological or pathological conditions, are poorly investigated. In this study, we first measured the 0.14 THz waves penetration across the skull of a C57BL/6 mouse and found the percentage of THz penetration to be ~70%, guaranteeing that THz waves can reach the relevant brain regions. We then exposed the APP^SWE^/PS1^DE9^ mouse model of AD to repeated low-frequency THz waves on the head. We demonstrated that THz waves treatment significantly improved the cognitive impairment and alleviated AD neuropathology including Aβ deposition and tau hyperphosphorylation in the AD mice. Moreover, THz waves treatment effectively attenuated mitochondrial impairment, neuroinflammation, and neuronal loss in the AD mouse brain. Our findings reveal previously unappreciated beneficial effects of THz waves treatment in AD and suggest that THz waves may have the potential to be used as a novel therapeutic intervention for this devastating disease.

**Supplementary Information:**

The online version contains supplementary material available at 10.1007/s12264-023-01145-3.

## Introduction

Alzheimer’s disease (AD) is the most common neurodegenerative disease worldwide [1]. As of 2019, more than 55 million people were affected by the disease worldwide, and this is projected to rise to 153 million by 2050 [2]. The growing prevalence of AD with aging has imposed enormous social and economic burdens on society [3]. Widespread evidence suggests that the overproduction and/or deficient clearance of amyloid-β (Aβ), leading to the cerebral accumulation of senile plaques [4], plays an essential role in the development of AD [5]. Overproduced and accumulated Aβ in the AD brain triggers subsequent pathological events such as tau-hyperphosphorylation, neuroinflammation, oxidative stress, neurite and synaptic degeneration, and neuronal loss [6–8]. These pathological events can form vicious cycles or networks to exacerbate disease progression [9, 10]. Lessons have been learned from currently failed clinical trials that pharmacological therapies targeting a single molecule or pathway are not sufficient to halt the progression of this complex disease [8, 11]. With the in-depth study of AD, non-pharmacological therapies including electromagnetic treatment have been emerging as a new intervention because of their promising therapeutic potential, mild side effects, and easy acceptance. The nervous system is generally sensitive to exogenous electromagnetic stimuli due to the bioelectric nature of neuronal activity. Furthermore, near-infrared therapy has been reported to have therapeutic benefits on retinal damage [12], brain aging [13], Parkinson’s disease [14], stroke [15], and depression [16]. Specifically for AD, previous studies have shown that near-infrared therapy has beneficial outcomes in animal models of AD [17, 18]. Moreover, AD transgenic mice exposed to electromagnetic treatment for 8-8.5 months show significantly reduced Aβ deposition in the hippocampus and cortex [19, 20]. In addition, using optogenetic and pulsed light irradiation methods, Iaccarino et al. demonstrated that 473 nm blue light irradiation can significantly reduce Aβ deposition in the visual cortex of a mouse model of dementia [21]. All these findings highlight the promising potential of electromagnetic therapy for the treatment of AD.

Terahertz (THz) waves are electromagnetic waves ranging between the microwave and infrared regions with a frequency of 0.1–10 THz and a wavelength of 0.03–3 mm [22]. The energy of THz waves is as low as a few millivolts, and the mechanism of their impact on living organisms is quite different from that of high-energy and potentially harmful irradiations, such as X-rays and gamma rays. THz technology has recently been used in the biomedical field, such as in the diagnosis of tumors and brain trauma [23–25]. THz waves have been shown to affect the nervous system *in vitro* [22, 26, 27]. For instance, THz waves at specific frequencies can change the permeability of nerve cell membranes [28]. Moreover, THz waves influence the gene expression of primary hippocampal neurons, including a total of 111 genes up-regulated and 54 downregulated [27]. By using different bioinformatics tools, such as differentially-expressed genes and differentially-expressed transcripts, and Gene Ontology or Kyoto Encyclopedia of Genes and Genomes enrichment, the results of a collective study revealed that the functions of these altered genes can be categorized into the regulation of neuronal growth, axonal genesis, and synaptic protein expression [27]. However, little is known about the biological effects of THz waves on AD.

In the present study, we used the APP^SWE^/PS1^DE9^ mouse model of AD and age-matched wild-type (WT) littermates to evaluate the possible beneficial impacts of THz waves on AD. AD or WT mice were exposed to chronic intermittent irradiation of low-frequency THz waves on the head for 3 months. The AD control and WT control mice were sham-treated without THz exposure. The spatial memory and AD pathology were assessed to evaluate the effects of the THz waves. Our data demonstrated for the first time that repeated THz waves exposure improved cognitive performance and alleviated AD pathology in AD transgenic model mice, including Aβ deposition and tau hyperphosphorylation. Moreover, THz waves also effectively attenuated mitochondrial impairment, neuroinflammation, and neuronal damage. Our findings reveal previously unappreciated beneficial effects of THz waves in AD and suggest the potential of THz waves as a novel strategy for AD therapy.

## Materials and Methods

### Animals

SPF-grade male 5-month-old APP^SWE^/PS1^DE9^ double mutant transgenic mice were purchased from the Jackson Laboratory (No. 005864) and their age- and gender-matched WT littermates were used as controls. The mice were allowed to adapt to the laboratory environment for at least 7 days before testing. The Animal Ethics Committee of Dalian Medical University approved the experimental protocols. As reported previously [29], APP^SWE^/PS1^DE9^ mice were genotyped by polymerase chain reaction amplification of genomic DNA extracted from tail snips with the following primers:

$$  \begin{aligned}    & {\text{5}}^{\prime } {\text{ - AGGACTGACCACTCGACCAG - 3}}^{\prime } \;{\text{(forward)}} \\     & {\text{5}}^{\prime } {\text{ - CGGGGGTCTAGTTCTGCAT - 3}}^{\prime } {\text{ (reverse)}}{\text{.}} \\  \end{aligned}  $$

### THz Exposure System

The setup of the THz exposure system is schematically illustrated in Fig. S1A. The THz waves were generated by a generator (RFPA S.A, Model RFS9001800-25), which can generate a pulse frequency of 0.14 THz at an average energy of 100 mW. A waveguide collected THz waves to amplify the power. An infrared thermal imager monitored the temperature changes of experimental animals. THz waves emitted by the THz source was fed into a horn antenna and collimated using a TPX lens to obtain a beam. The optical fiber transmitted the THz waves to the forehead of the experimental animal. The transmission efficiency of the fiber was 10%, which meant that the power reaching the tail end of the fiber was 10 mW. The diameter of the optical fiber was <1 mm. Considering that the optical fiber initially radiated to the mouse head with a certain degree of divergence, the optical spot was assumed to be 5 mm (upper limit), so the irradiation area was 19.63 mm^2^ (~0.1963 cm^2^). For the convenience of calculation, this was taken to be 0.2 cm^2^. The power density was:

$$ {1}0\;{\text{mw/}}0.{2}\;{\text{cm}}^{{2}} = {5}0\;{\text{mW/cm}}^{{2}} . $$

Considering the pulse factor, the final power density of the fiber terminal was 25 mW/cm^2^. Power/energy studies have shown that a therapeutic dose of 4 J/cm^2^ is optimal in some cases [17]. About 6 J/cm^2^ is equivalent to using a 30 mW/cm^2^ LED device with a duration of 200 s. More powerful equipment could mean shorter treatment times. In this experiment, 10 min was finally chosen to irradiate mice.

### Skull Penetration Tests and Biosafety Measurement of Terahertz Waves

The transmission curve of mouse calvaria was measured by THz-TDS (time-domain spectroscopy). The THz-TDS is a commercially available THz spectral system (Zomega, Z3-XL), pumped with a Ti: Sapphire femtosecond laser (Coherent, Vitara). The THz pulses are produced in the standard way by using a semiconductor dipole antenna. In the measurement process, the calvaria were held in a sample holder, and then the THz irradiation was introduced to pass through the calvaria and entered an electro-optic sampling module to complete the conversion of photoelectric signals. The percentage of THz waves penetration across the skull of a C57BL/6 mouse was ~70%, which guarantees that THz can penetrate calvaria and act on the relevant brain regions (see Fig. S1B). We evaluated the biosafety issue of THz waves in WT mice after 12 weeks of THz treatment. The evaluation criteria included behavioral performance, body weight, morphological changes of neurons, neuroinflammation, and mitochondrial function in the brain of WT mice with or without THz treatment.

### Behavioral Tests

**Fig. 1:**
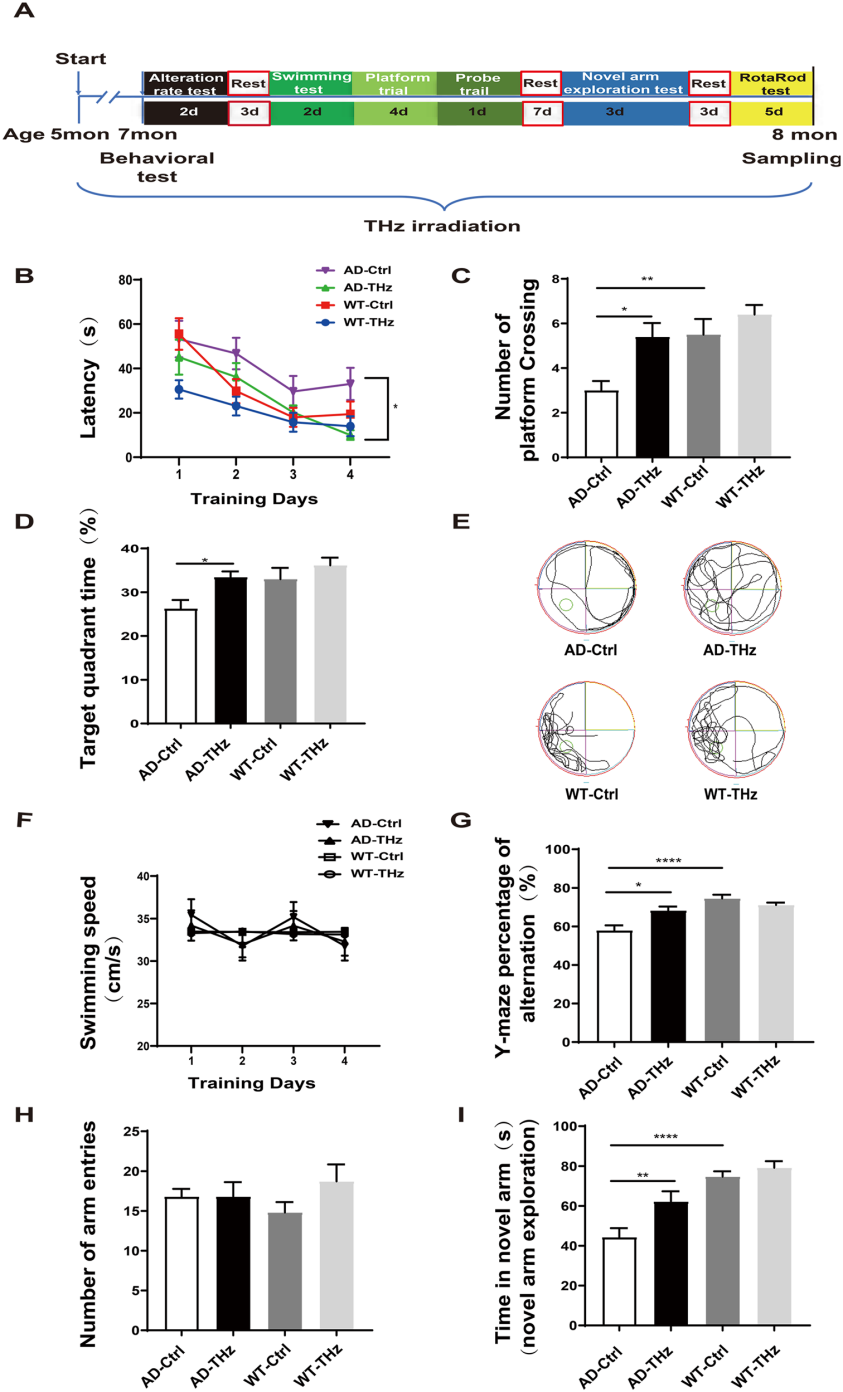
THz waves improve behavioral performance in APP^SWE^/PS1^DE9^ mice. **A** Experimental schematic of THz irradiation and behavioral tests. **B** Escape latency in the Morris water maze (MWM). **C** Number of crossings through the target quadrant (where the platform was previously located) in the probe test of the MWM. **D** Time in the target quadrant of the MWM after removing the platform. **E** Images of mouse trajectories in the MWM recorded by computer. **F** Mean swimming speed of each group in the MWM. **G–I** Y-maze test; *n =* 10 per group. Data are presented as the mean ± SEM. ****P <0.0001, **P <0.01, *P <0.05.

As described previously [30–32], mice underwent multiple behavioral tests in the order of Y-maze, Morris water maze, and Novel arm exploration test. A 3- or 7-day interval was set for different types of behavioral tests (Fig. 1A). In order to avoid the influence of stress factors on the experimental results, we placed the mice in the behavioral laboratory one week before tests to make the mice familiar with the laboratory environment. In the spontaneous alternation test, mice were allowed to move freely through a Y-maze during a 5-min session. Alternation was defined as successful entries into the three arms on overlapping triplet sets. The percentage of alternation was calculated as the total number of alternations × 100 / (total number of arm entries − 2). The novel arm exploration test was also applied in a Y-maze decorated with a different pattern after the Morris water maze. Before the Morris water maze test, the swimming ability of all mice was tested. The Morris water maze test consisted of three platform trials per day for 4 consecutive days, followed by a probe trial. The escape latency was measured in platform trials. The annulus crossings and the time spent in each quadrant were measured in probe trials. All of the behavioral parameters were tracked, recorded, and analyzed using SMART 3.0 software (Harvard Apparatus).

### ELISA

Each frozen brain was homogenized in liquid nitrogen, and part of the resultant powder was extracted. The concentrations of Aβ42, Aβ40 (Elabscience, E-EL-H0542c to detect Aβ40, and E-EL-H0543c to detect Aβ42), IL-6, and TNF-α (Animal Union, Shanghai, China) in brain extracts were determined using ELISA kits according to the manufacturer’s instructions.

### Western Blotting

Mice were sacrificed after the behavioral tests, the tissues were dissected rapidly on ice, homogenized in cold RIPA buffer (Beyotime Biotechnology, Shanghai, China) containing protease and phosphatase inhibitor cocktails (Sigma-Aldrich, St. Louis, MO, USA), and then lysed for 30 min on ice. The protein concentration in the supernatant was determined using protein assay kits (TaKaRa, Shiga, Japan). Forty micrograms of protein were loaded and separated by sodium dodecyl sulfate/polyacrylamide gel electrophoresis and then transferred to polyvinylidene fluoride membranes (Millipore, Bedford, MA, USA). After blocking, the membranes were incubated with appropriate primary antibodies (Table S1) at 4°C overnight, followed by 1 h incubation at room temperature with a peroxidase-conjugated secondary antibody. Finally, the membrane was incubated with enhanced chemiluminescence (Millipore, Bedford, MA, USA), and the target protein bands were quantified using the FluorChem Q system (ProteinSimple, CA, USA).

### Immunofluorescent and Thioflavin S (ThioS) Staining

After the behavioral tests, the mice were anesthetized and transfused with phosphate-buffered saline (PBS; Boster, China). Each brain was quickly removed and post-fixed with 4% paraformaldehyde overnight. The brains were then incubated in 30% sucrose in PBS for cryoprotection, and 40 μm serial sections were cut on a Leica cryostat (CM-1950S, Leica, Germany). The sections were next incubated with blocking buffer (10% normal goat serum, 1% bovine serum albumin, and 0.3% Triton X-100, PBS solution) overnight at 4°C and then incubated with the primary antibodies overnight at 4°C (Table S1). The stained sections were imaged using a laser scanning confocal microscope (A1 confocal, Nikon Instruments (Shanghai) Co., Ltd). The paired images in the figures were collected at the same gain and offset settings. For ThioS staining, sections were stained with 0.05% ThioS (23059, AAT Bioquest) in 50% ethanol in the dark for 8 min at room temperature, followed by two rinses in 80% ethanol for 10 s each. Immunofluorescence images were captured using a fluorescent microscope (Olympus) and analyzed using ImageJ software.

### Transmission Electron Microscopy

As described previously [33], mice were sacrificed after the behavioral tests. The CA1 area of the hippocampus was collected rapidly on ice within 3 min into a fixative solution containing 2.5% glutaraldehyde (Servicebio, Wuhan, China) and then fixed at room temperature for 2 h followed by transfer to 4°C for storage. The tissue was washed three times in PBS (pH 7.4) before post-fixing in 1% osmium acid (diluted with 0.1 mol/L PBS solution) at room temperature for 2 h and was successively dehydrated. After a series of embedding steps, the tissue was cut into 80-nm sections using the Leica ultrathin microtome (Leica UC7, Leica, Germany) and stained with 2% uranyl acetate saturated alcohol solution and lead citrate solution, respectively. Finally, the stained sections were imaged using a transmission electron microscopy (TEM, HITACHI, HT7700).

### Statistical Analysis

The results were presented as the mean ± SEM. As applicable, statistical comparisons between the two groups were made using the Student’s *t*-test or the Mann–Whitney U-test. One-way ANOVA and Tukey’s test were used to compare four groups. P values <0.05 (two-sided) were considered significant. All analyses were performed with GraphPad Prism software, version 9.0.

## Results

### Impact of THz Treatment on Cognitive Function in AD Mice

To investigate the effects of THz waves on cognitive impairment in AD, we conducted THz intervention experiments in APP^SWE^/PS1^DE9^ mice. The experimental schematic is described in Fig. 1A. WT and AD mice at 5 months of age were each randomly divided into two subgroups: WT control, WT-THz, AD control, and AD-THz, with 18 mice in each group. Animals in the AD-THz and WT-THz groups were exposed to low-frequency THz waves (0.14 THz, 10 min per day at 20:00, 5 days per week for 12 consecutive weeks). The mice were exposed to THz waves for 3 months starting from 5 months of age. All mice were analyzed at 8 months of age because AD mice begin to develop amyloid plaques at 6–7 months of age and a spatial learning deficit at 7 months of age [34]. As expected, our results showed that AD control mice (8 months old) displayed a significant cognitive decline in terms of spatial learning compared with the age-matched WT control mice (70% longer latency *vs* WT control on the fourth day of training; Fig. 1B). In addition, the mice in the AD-THz group presented an improved spatial memory, as shown by a significantly shorter escape latency than AD control mice (71% reduction of latency in 8-month-old AD-THz mice *vs* AD control; *P <*0.05; Fig. 1B).

To determine the effect of THz waves on spatial memory consolidation, probe trials were applied to assess the maintenance of spatial memory. Compared with WT control mice, AD control mice crossed the target quadrant less often and spent less time in the target quadrant (45% reduction; *P <*0.01) in crossing numbers and 20% reduction of time in the target quadrant; Fig. 1C, D), indicating an impaired memory consolidation. After THz exposure, AD-THz mice spent significantly more time in the target quadrant and showed increased crossings compared with AD control mice (80% increase in crossing times and 27.3% longer time spent in the target quadrant, both *P <*0.05; Fig. 1C, D), suggesting an improved cognitive function. No difference was found in swimming speed during platform trials among all groups (Fig. 1F).

Consistently, in the Y-maze test, compared with WT control mice, AD control mice showed a relatively lower alternation rate and less time spent in the novel arm (22% reduction in alternation rate and 40.7% less time in the novel arm, both *P <*0.0001; Fig. 1G, I). However, THz-exposed AD mice showed a relatively higher alternation rate and more time spent in the novel arm compared with AD control mice (18% increase, *P <*0.05) in alternation rate and 40.5% longer time (*P <*0.01) in the novel arm; Fig. 1), implying an ameliorating effect of THz exposure on the cognitive impairment in AD. The exploratory behavior was intact among all groups, as evidenced by the unchanged total arm entries (Fig. 1H). In addition, the rotarod test showed no significant difference between AD and WT mice in the latency and maximum speed of falling (Fig. S2), indicating that the motor and balance abilities of mice were intact after THz treatment.

### THz Treatment Alleviates Aβ Pathology and Tau Hyperphosphorylation in AD Mice

**Fig. 2:**
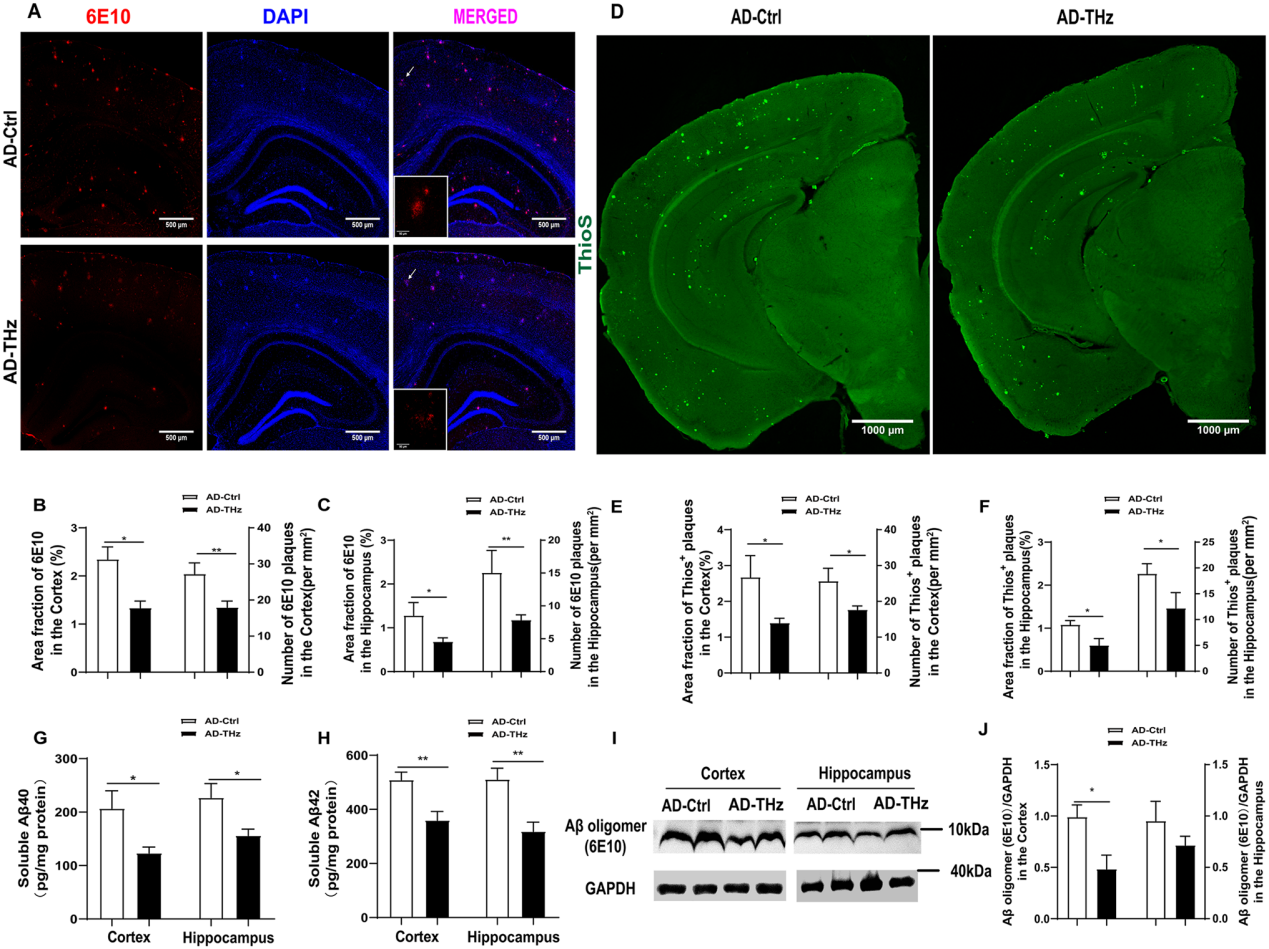
THz waves attenuate Aβ pathology in the brain of APP^SWE^/PS1^DE9^ mice. Representative images of 6E10 immunofluorescence staining in AD Ctrl and AD-THz groups. Insets: representative morphology at a higher magnification. Scale bars, 500 µm, 50 µm. **A–C** Comparison of the 6E10 and Aβ plaques in the cortex and hippocampus (Hip). *n =* 6 per group. **D–F** Comparison of the Thios^+^ plaques in the cortex and hippocampus (Hip). Scale bars, 1000 µm, *n =* 5 per group. **G, H** Comparison of Aβ40 and Aβ42 levels measured by ELISA in brain homogenates. Aβ levels are normalized by total protein concentration. **I, J** Protein levels of Aβ oligomer with 6E10 antibodies detected by Western blots (*n =* 3 per group). Data are presented as the mean ± SEM. **P <0 .01, *P <0.05.

Aβ plaques are considered the primary pathological hallmark of AD, and Aβ load is often used as a biomarker for AD severity [35, 36]. In our study, Aβ immunofluorescence staining was performed using the 6E10 antibody to quantify Aβ plaques. The AD control mice showed abundant Aβ plaque deposition in the cortex and hippocampus at 8 months of age (Fig. 2A). Remarkably, compared with AD control mice, the AD-THz mice displayed a significant amelioration of Aβ pathology, as evidenced by the reduced area fraction and plaque density of Aβ plaques in both the cortex (43% reduction in area fraction, *P <*0.05; 34% reduction in plaque density, *P <*0.01; Fig. 2B) and hippocampus (46% reduction in area fraction, *P <*0.05; and 47% reduction in plaque density, *P <*0.01; Fig. 2C). Similarly, ThioS staining revealed a significant decrease in the area fraction and plaque density of insoluble Aβ plaques in both the cortex (47.9% reduction in area fraction, *P <*0.05; 31.2% reduction in plaque density, *P <*0.05; Fig. 2E) and hippocampus (44 % reduction in area fraction, *P <*0.05; 35.4% reduction in plaque density, *P <*0.05; Fig. 2F) of AD-THz mice, compared with AD control mice. Consistent with these data, ELISA showed significantly lower levels of Aβ_42_ and Aβ_40_ in the brain homogenates from AD-THz mice, compared to AD controls (40% reduction of Aβ40, *P <*0.05; 29% reduction of Aβ42, *P <*0.01) in the cortex. In the hippocampus, there was a 31% reduction of Aβ40 (*P <*0.05) and a 38% reduction of Aβ42 (*P <*0.01) (Fig. 2G, H). These data demonstrate that THz treatment can reduce Aβ levels and suppress the formation of Aβ plaques in the AD brain.

Next, we measured soluble neurotoxic Aβ oligomers with Western blot using 6E10 antibody (Fig. 2I). Similar to immunostaining results, the 6E10 immunopositive blots of Aβ oligomers ~10 kDa were significantly reduced in the cortex of AD-THz mice (*P <*0.05; Fig. 2I, J) and moderately reduced in the hippocampus of AD-THz mice (*p*>0.05; Fig. 2I, J), compared with AD control mice.

**Fig. 3:**
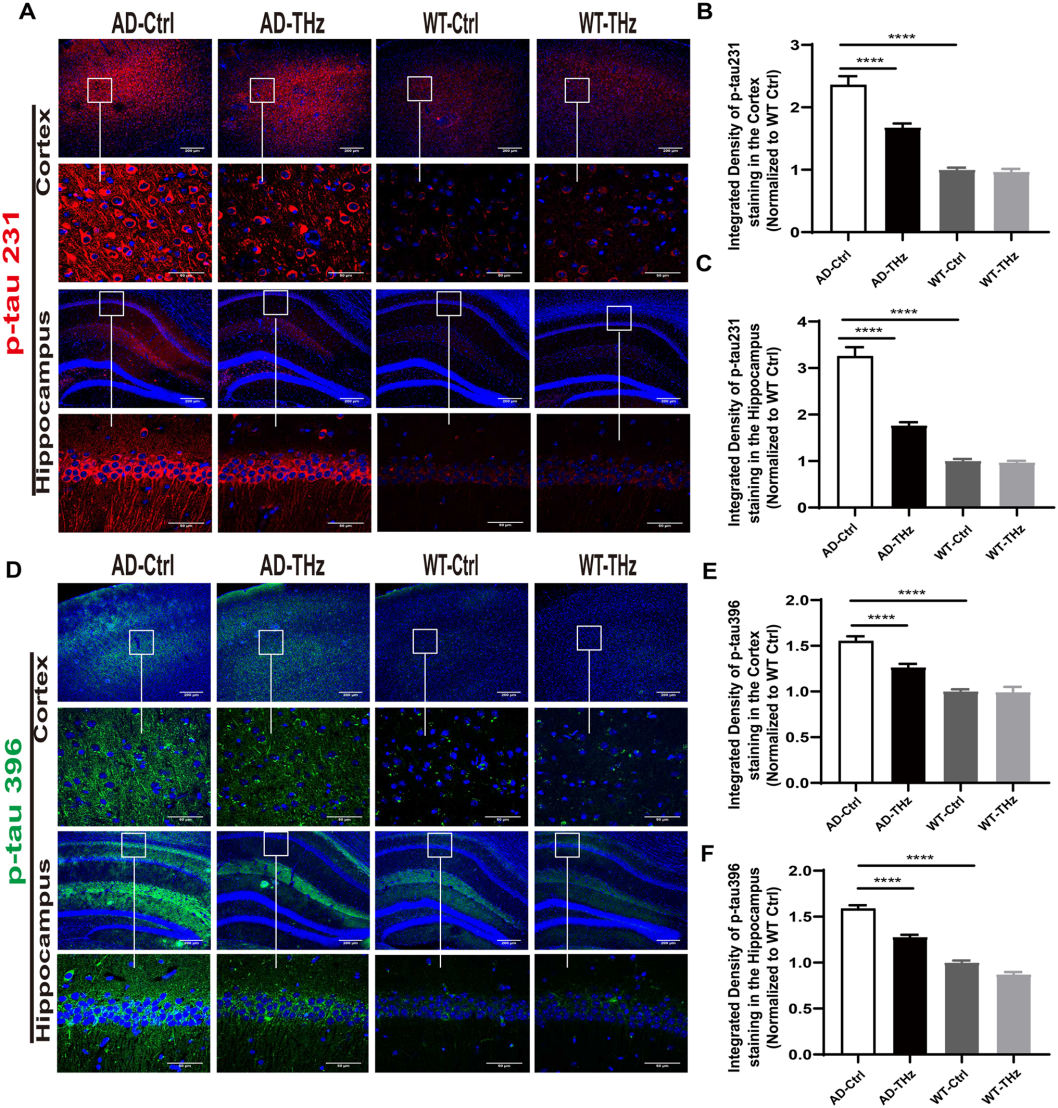
THz waves attenuate tau hyperphosphorylation in the brain of APP^SWE^/PS1^DE9^ mice. Tau phosphorylation was evaluated by immunofluorescence staining. **A–F** Representative images and quantitative analysis of p-tau 231 and p-tau 396 positive staining in the cortex and hippocampus (*n =* 6 per group), Insets: representative morphology at a higher magnification. Scale bars, 200 μm, 50 μm. ****P < 0.0001.

We next measured the effects of THz waves on tau hyperphosphorylation, which is another key event in AD, resulting in the formation of neurofibrillary tangles as the second pathological hallmark of AD [37, 38]. Compared with the WT control mice, AD control mice showed much more disease-related phospho-tau (p-tau) 231 and p-tau 396 positive neurons in the cortex and hippocampus than WT control mice (136% and 55% increase of p-tau 231 and p-tau 396 positive neurons in the cortex, respectively, both *P <*0.0001; 225% and 58% increase of p-tau 231 and p-tau 396 positive neurons in the hippocampus, respectively, both *P <*0.0001; Fig. 3A-F). However, the disease-related p-tau 231 and p-tau 396 positive neurons in the cortex and hippocampus of THz-treated AD mice were considerably less than those of AD control mice (29% and 19% reduction of p-tau 231 and p-tau 396 positive neurons in the cortex, both *P <*0.0001; 45% and 20% reduction of p-tau231 and p-tau 396 positive neurons in the hippocampus, both *P <*0.0001; Fig. 3A-F).

**Fig. 4:**
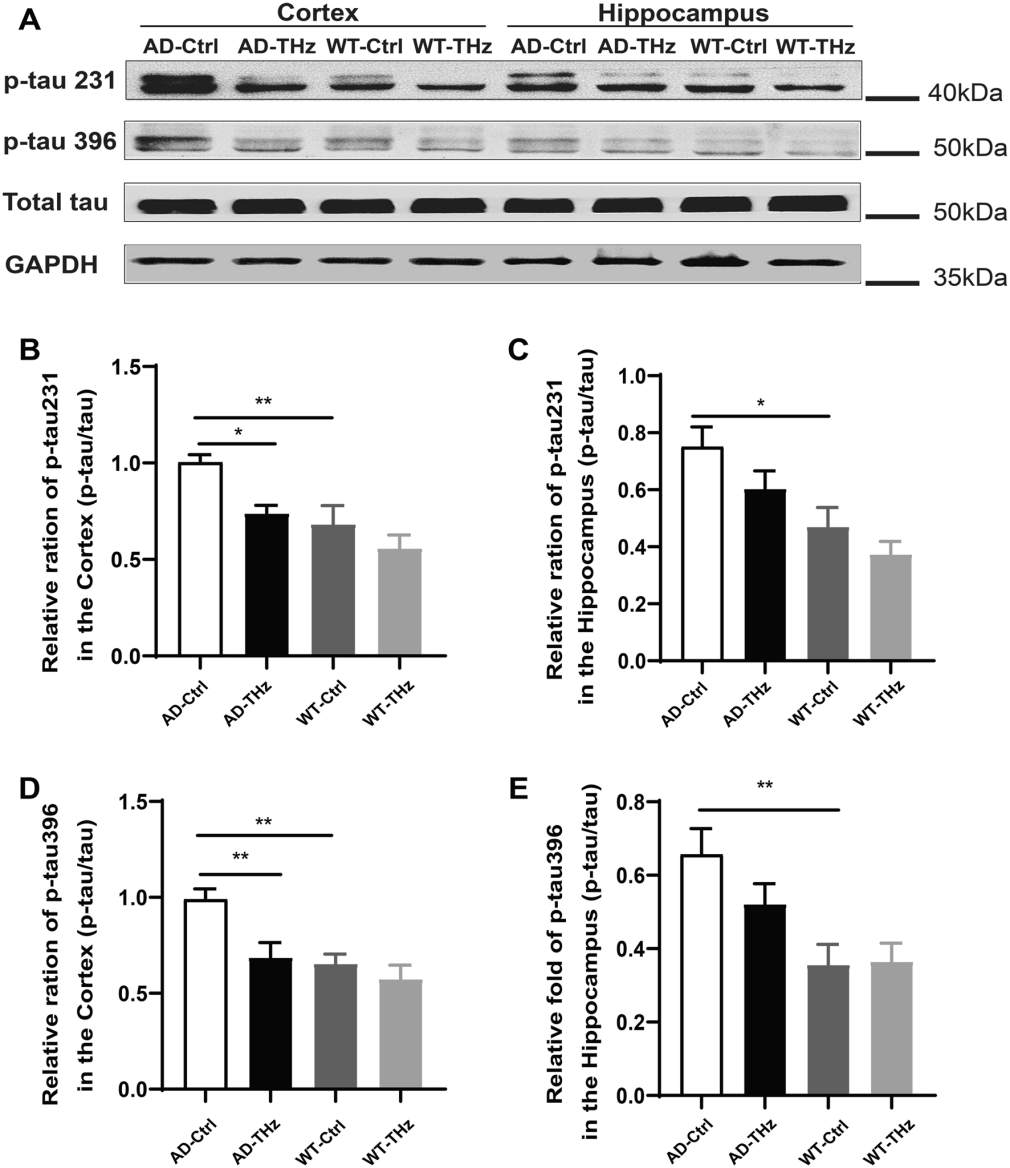
THz waves attenuate tau hyperphosphorylation in the brain of APP^SWE^/PS1^DE9^ mice.** A–E** Protein expression of p-tau 231 and p-tau 396 assessed by Western blots (*n =* 3 per group). Data are presented as the mean ± SEM. **P <0.01, *P <0.05.

Western blots further confirmed that the higher levels of tau hyperphosphorylation (47.2% and 51.4% increase of p-tau 231 and p-tau 396, both *P <*0.01) in AD control mice than those of WT control mice in the cortex (Fig. 4B, 4D). There were 60% (*P <*0.05) and 84.4% (*P <*0.01) increases of p-tau 231 and p-tau 396, respectively, in the AD control mice *vs* WT control mice in the hippocampus (Fig. 4C, E). Western blots also showed that THz waves attenuated tau hyperphosphorylation (26.4% reduction of p-tau 231, *P <*0.05; 30.7% reduction of p-tau 396, *P <*0.01; Fig 4B, D) in the AD-THz mice *vs* AD control mice in the cortex. p-tau levels were moderately reduced in the hippocampus of AD-THz mice (*P* >0.05; Fig 4C, E), compared with AD control mice. These results indicate that 3 months of repetitive THz exposure may shield the AD brain against tau hyperphosphorylation.

### THz Exposure Attenuates Neuronal and Dendritic Loss in AD Mice

**Fig. 5:**
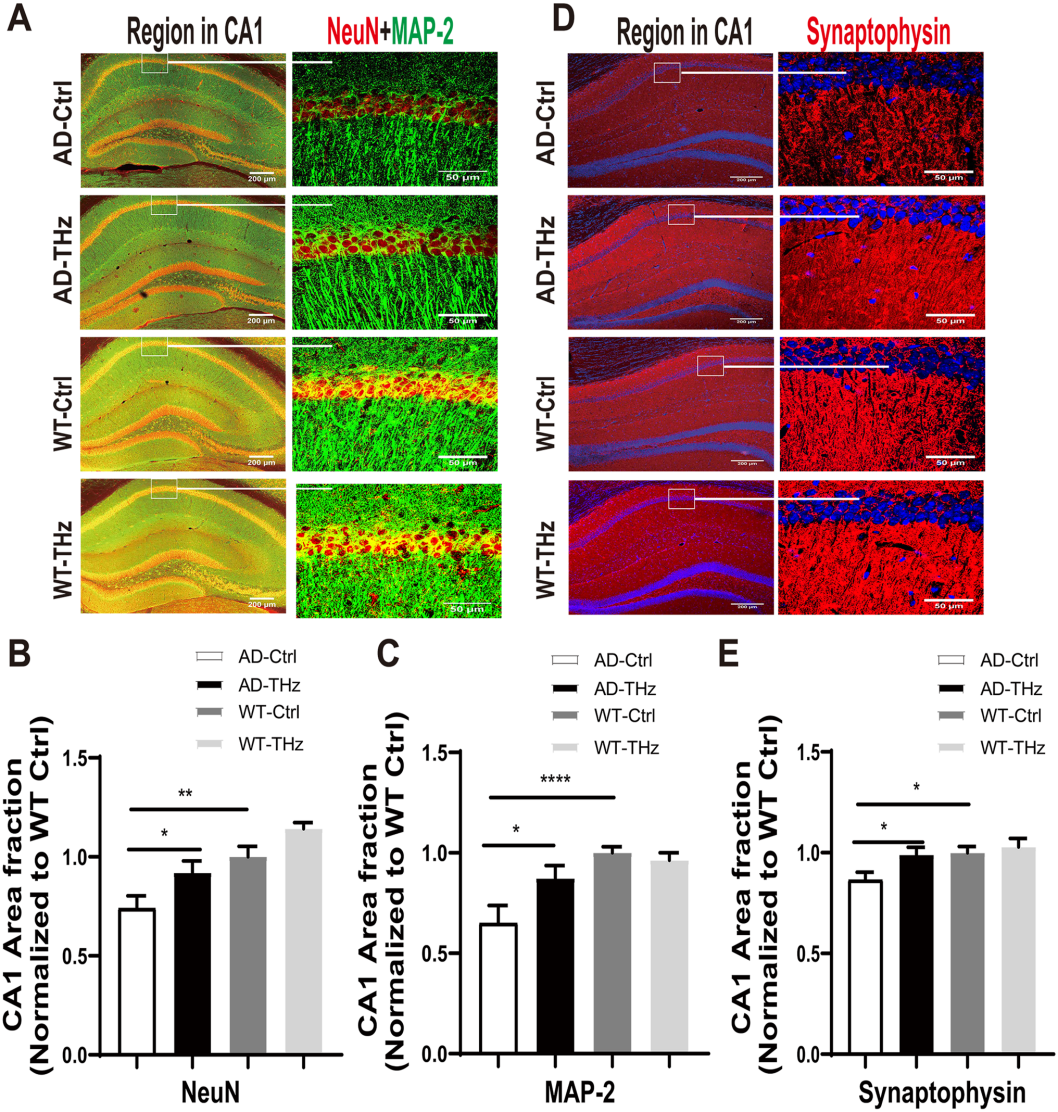
THz waves rescue neuronal and dendritic loss in the brain of APP^SWE^/PS1^DE9^ mice. Representative images (**A, D**) and quantification (**B–E**) of positive staining area fractions of neurons and dendrites in the region of the hippocampus stained by anti-NeuN (red) and anti-MAP-2 (green) or anti-synaptophysin (red) antibodies, *n =* 4-5 per group. Insets: representative morphology at higher magnification. Scale bars, 200 μm, 50 μm. Data are presented as the mean ± SEM. ****P <0.0001, **P <0.01, *P <0. 05. AD. APP^SWE^/PS1^DE9^ transgenic mice; Ctrl, control; WT. wild type.

Neuronal degeneration is an event downstream of Aβ oligomer toxicity. To further examine the potential effect of THz on neurons in the AD brain, we applied immunofluorescence staining using antibodies against NeuN, microtubule-associated protein 2 (MAP-2), and synaptophysin in the hippocampus of THz-treated mice. We found that the AD mice at 8 months old showed a significant neuronal and dendritic loss in the hippocampus compared to WT control mice (25.9% reduction of NeuN, *P <*0.01; 35% reduction of MAP-2, *P <*0.0001; 13.5% reduction of synaptophysin, *P <*0.05; Fig. 5B, C, E), whereas THz waves exposure remarkably reversed the neuronal and dendritic loss in the AD-THz mice compared to the AD control mice, as shown by the changes of positive staining area fraction (24.1%, 34.2%, and 14.4% increase of NeuN, MAP-2, and synaptophysin, respectively, all *P <*0.05; Fig. 5B, C, E). The THz treatment in WT mice did not produce any significant changes in the positive staining area fractions of NeuN, MAP-2, and synaptophysin (Fig. 5A, D). These results demonstrated that THz waves can ameliorate neuronal loss in the hippocampus of AD mice.

### THz Exposure Reduces Neuroinflammation in the Brain of APP^SWE^/PS1^DE9^ Mice

**Fig. 6:**
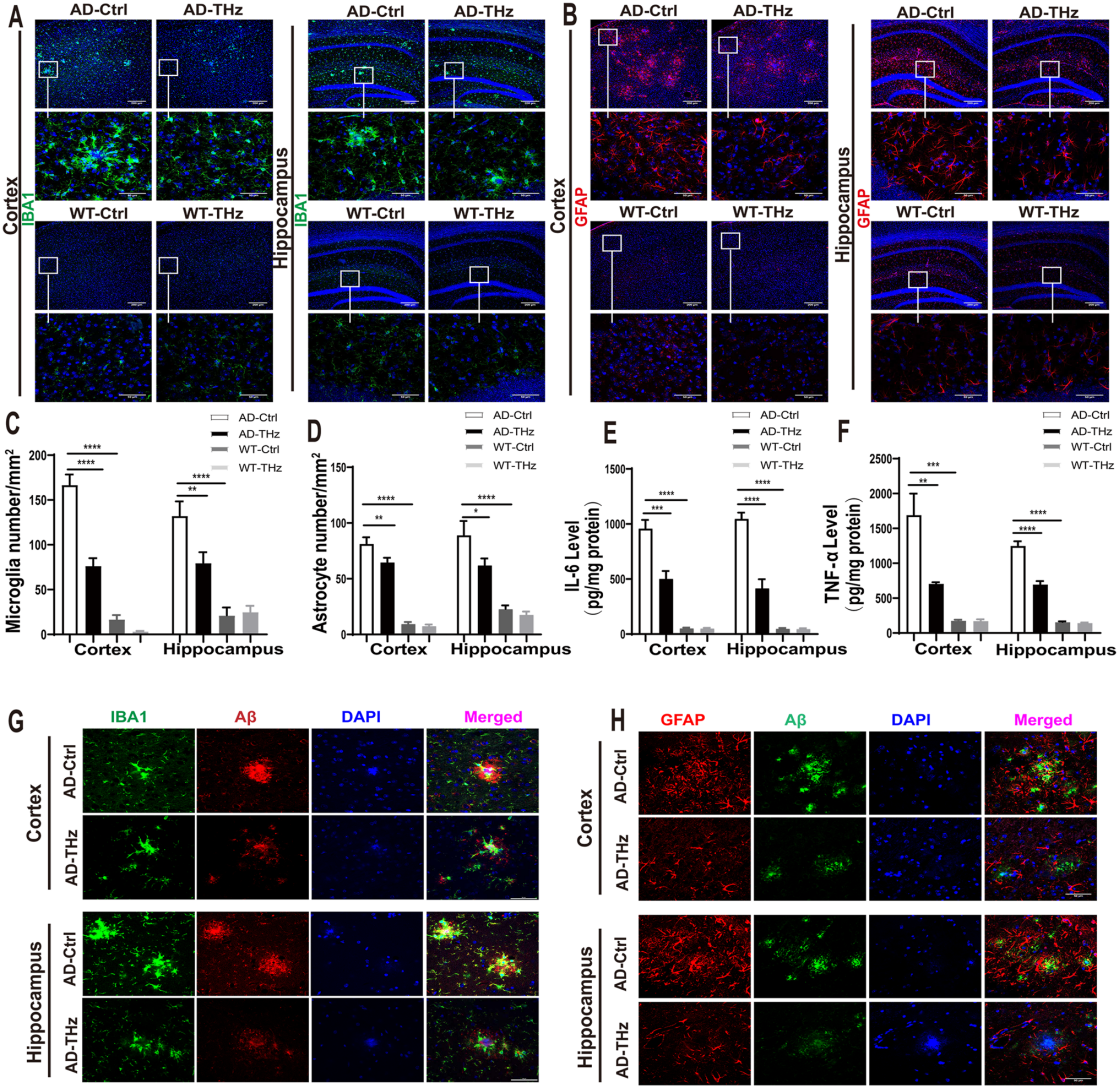
THz waves suppress neuroinflammation in the brain of APP^SWE^/PS1^DE9^ mice. THz waves decrease glial reactivity in APP^SWE^/PS1^DE9^ mice. **A** Representative immunostaining of IBA-1 (green) in the cortex and hippocampus (Hip) of the different groups. Scale bars, 200 µm, 50 µm. **B** Representative immunostaining of GFAP (red) in the cortex and hippocampus of the different groups. Insets: representative morphology at higher magnification. Scale bars, 200 µm. **C** Numbers of microglia in the cortex and hippocampus. **D** Numbers of astrocytes in the cortex and hippocampus. *n =* 6 mice per group for AD, *n =* 3 mice per group for WT. **E, F** THz waves decrease the of production pro-inflammatory cytokines IL-6 and TNF-α in the brain of APP^SWE^/PS1^DE9^ mice, measured by ELISA. *n =* 6 per group for AD, *n =* 4 mice per group for WT. **G** Colocalization of fluorescent Aβ (red), Iba1 (green), and DAPI (blue) in the cerebral cortex and hippocampus. Scale bar, 50 μm. **H** Colocalization of fluorescent Aβ (green), GFAP (red), and DAPI (blue) in the cerebral cortex and hippocampus. Scale bar, 50 µm. Data are presented as the mean ± SEM. ****P <0.0001, ***P <0.001, **P <0.01. AD APP^SWE^/PS1^DE9^, transgenic mice; Ctrl, control; WT. wild type.

Neuroinflammation has been reported to be involved in the pathogenesis and progression of AD, as evidenced by the presence of activated microglia and astrocytes surrounding Aβ plaques [39]. To investigate the anti-inflammatory potential of THz waves on AD mice, we quantified the microglia and astrocytes in the cortex and hippocampus by immunofluorescent staining using ionized calcium-binding adapter molecule 1 (IBA1) and glial fibrillary acidic protein (GFAP) antibodies, respectively. The staining revealed marked astrogliosis and microgliosis in the cortex and hippocampus of AD control mice compared with WT controls, as evidenced by the increased fluorescence intensity. The counts of microglia and astrocytes were significantly higher in both the cortex and hippocampus of AD control mice than those in age-matched WT control mice (884.7% and 755% increase of microglia and astrocytes in the cortex, both *P <*0.0001; 552% and 289% increase of microglia and astrocytes in the hippocampus, both *P <*0.0001; Fig 6A-D). Moreover, the counts of microglia and astrocytes were significantly downregulated in the THz-treated AD mice compared with those in AD control mice; reduction of microglia (54%, *P <*0.0001) and astrocytes (20%, *P <*0.01) in the cortex (Fig. 6A-D); and reduction of microglia (40%, *P <*0.01) and astrocytes (30%, *P <*0.05) in the hippocampus (Fig 6A-D). We also observed that Aβ plaques were surrounded by reactive microglia and astrocytes in the brains of AD control mice, whereas reactive microglia and astrocytes were reduced around Aβ plaques in the brains of AD-THz mice (Fig. 6G, H). Moreover, the THz waves did not enhance Aβ uptake in microglia in the brain (see Fig. S3).

We further measured the levels of proinflammatory cytokines in different groups of mice. Consistent with IBA1 and GFAP staining, our results demonstrated that the significantly higher levels of interleukin-6 (IL-6) (increased by 1662% and 1965%, respectively, both *P <*0.0001) and tumor necrosis factor-α (TNF-α) (increased by 843% and 686%, respectively, both *P <*0.001) in the cortex and hippocampus in the AD control mice compared to those of WT control mice (Fig. 6E, F). Moreover, THz exposure in the AD-THz mice significantly reduced the levels of these proinflammatory molecules in both the cortex (47%, reduction of IL-6, *P <*0.001; 58% reduction of TNF-α, *P <*0.01) and hippocampus (60% reduction of IL-6, and 44% reduction of TNF-α in the hippocampus, both *P <*0.0001) as compared to the AD control mice (Fig. 6E, F), suggesting an ameliorating effect of THz waves treatment on the neuroinflammation in AD mouse brain.

### THz Exposure Modulates Mitochondrial Dysfunction in the Brain of APP^SWE^/PS1^DE9^ Mice

**Fig. 7:**
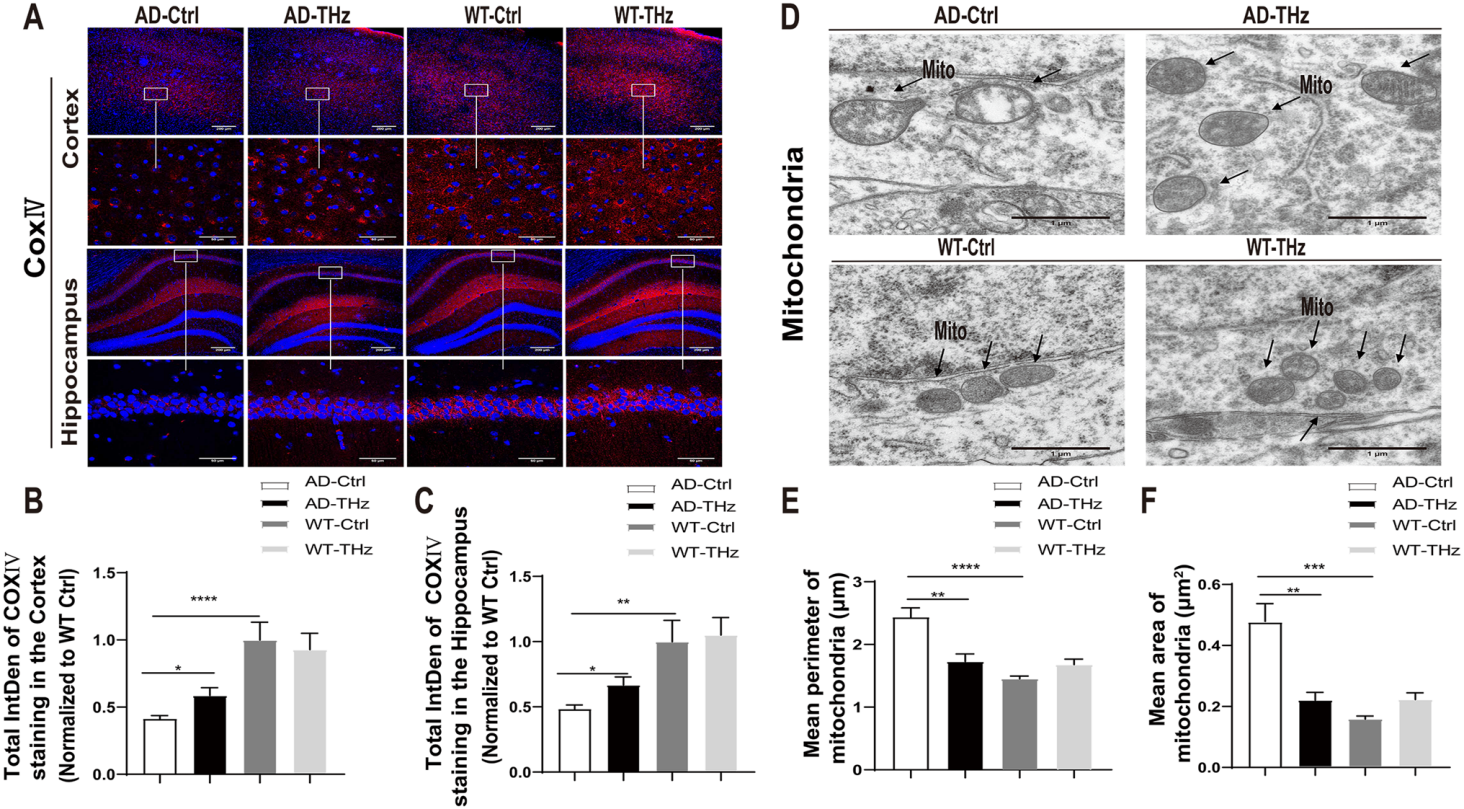
THz waves mitigate mitochondrial dysfunction in the brain of APP^SWE^/PS1^DE9^ mice. **A** Effect of THz waves treatment on cytochrome oxidase expression (red) in the cortex and hippocampus of different groups of mice. Insets: representative morphology at higher magnification. Scale bars, 200 µm, 50 µm. **B, C** Total COX-IV fluorescence intensity in different brain regions (*n =* 4-5 mice per group). **D** Representative TEM images of mitochondria. Scale bars, 1 μm. **E** Mean perimeter of mitochondria, **F** Mean area of mitochondria (*n =* 3 mice per group). Data are presented as the mean ± SEM. ****P <0.0001, ***P <0.001, **P <0.01, *P <0.05. AD APP^SWE^/PS1^DE9^, transgenic mice; Ctrl, control; WT. wild type, IntDen. integrated density; Mito. mitochondria.

Accumulating evidence indicates that Aβ oligomer-induced mitochondrial dysfunction is one of the early pathological features of AD [40, 41]. In order to evaluate the modulating potential of THz treatment on mitochondrial dysfunction in AD mouse brain, we assessed the expression of cytochrome c oxidase (COX), a marker of mitochondrial function in the cortex and hippocampus, using an antibody against COX subunit IV (COX-IV). AD control mice showed a significantly decreased intensity of COX-IV in the cortex (59% reduction, *P <*0.0001) and hippocampus (52% reduction, *P <*0.01) compared to WT control mice (Fig. 7B, C). In addition, the THz-exposed AD mice showed a significantly increased intensity of COX-IV in the cortex and hippocampus (42% and 38% increase in the cortex and hippocampus, respectively, both *P <*0.05) compared to AD control mice (Fig 7B, C), indicating a considerable recovery of mitochondrial function following THz waves treatment.

TEM was also used to assess the mitochondrial morphology in the CA1 region of the hippocampus. Compared to the WT mice, large and vacuolate mitochondria were found in the hippocampus of AD control mice (Fig. 7D). Moreover, the mean perimeter and area of the mitochondria were dramatically increased by 67% (*P <*0.0001) and 206% (*P <*0.001), respectively, in the AD control mice compared to WT control mice (Fig. 7E, F), indicating mitochondrial swelling. Interestingly, THz waves treatment in THz-AD mice significantly attenuated the mean perimeter (reduced by 29%, *P <*0.01) and area of the mitochondria (reduced by 54%, *P <*0.01) in the hippocampus, respectively, compared to AD control mice (Fig. 7E, F).

### Penetration and Biosafety Assessment of THz Irradiation Apparatus Developed for Non-invasive Treatment

A THz irradiation apparatus was developed for the non-invasive treatment of APP^SWE^/PS1^DE9^ mice. Figure S1 A provides a visual representation of the apparatus, while the "Methods" section explains its structure in detail. During the irradiation process, the mice were awake and securely immobilized in the apparatus, ensuring that the treatment was delivered accurately and with minimal discomfort. We used a THz frequency of 0.14 THz, which is towards the lower end of the THz frequency range. The duty cycle was 50%, which refers to the amount of time that the irradiation is on compared to the total cycle time. To transmit the THz waves, a dielectric-metal fiber was utilized. This type of fiber is specially designed to allow for the efficient and low-loss transmission of THz waves over long distances. It consists of a metal coating on the inside and a non-conductive dielectric material on the outside. The THz-TDS measurements revealed that ~70% of the incident power of 0.14 THz waves was able to penetrate the skull (shown in Fig. S1). This indicated that THz irradiation had the potential to reach the designed brain regions when applied to the scalp. Specifically, when an incident irradiance of 25 mW/cm^2^ was used, the irradiance that reached the brain was estimated to be ~17 mW/cm^2^.

Biosafety concern is a prerequisite for THz irradiation’s future use in the bioengineering field, so we evaluated the safety issue of THz waves in WT mice. After 12 weeks of THz exposure, WT mice showed no significant alterations in behavioral tests (Fig. 1). In terms of neuronal morphology and synaptic density, no significant difference was found between WT-THz and WT-control mice (Fig. 5), which was consistent with the results of behavioral tests. Moreover, no alteration in neuroinflammation and mitochondrial function was found between WT-THz and WT-control mice (Figs 6, 7). In addition, we measured the body weight of mice before and after THz treatment, showing no significant difference among these four groups of mice (Fig. S4). Together. these data strongly suggest the safety of the THz waves used in this study.

## Discussion

AD is a neurodegenerative disease characterized by progressive memory deficits and cognitive decline [42], together with Aβ deposition, tau hyperphosphorylation, elevated oxidative stress and neuroinflammation, mitochondrial dysfunction, synaptic loss, and neurodegeneration in the brain [43]. Numerous efforts have been attributed to the development of non-pharmacological therapy against the cognitive decline in AD [44, 45]. Since the nervous system is more sensitive to exogenous electromagnetic irradiation [46, 47], special frequency electromagnetic waves have been applied to rescue cognitive deficits and AD neuropathology in AD mice [21, 45, 48]. In this study, for the first time, we discovered the promising potential of repeated low-frequency THz waves to effectively alleviate cognitive deficits and AD neuropathology in APP^SWE^/PS1^DE9^ mice. We demonstrate that low-frequency THz waves intervention for three months can rescue the cognitive function and AD neuropathology in AD mice, which provides a new concept and experimental basis for the early prevention and treatment of AD.

Over the last decade, the wide expansion of THz technology has reinvigorated interest in exploring the biological effects of THz waves [49]. The photon energy of the THz waves (0.004-0.04 eV) is only one-millionth of that of X-rays and seems not to cause tissue damage or side effects [28, 50]. Here, we evaluated the safety of THz waves after exposure to the mouse brain. The WT mice exposed to the THz waves for three months showed no significant alterations in behavioral performance, neuronal morphology, mitochondrial function, and synaptic density. Importantly, the THz waves also had a beneficial effect on cognitive performance and dramatically improved learning and memory in AD mice (Fig. 1). Note that we only tested the impact of relatively short-term exposure (five days per week for 12 weeks) of low-frequency THz waves in AD mice. Considering that AD is a chronic and progressive neurodegenerative disease, long-term exposure to low-frequency THz waves might be considered in the future to evaluate the long-lasting beneficial effects of THz waves on the symptoms and neuropathology of AD. Therefore, our findings provide a basis for THz treatment as a safe and effective strategy for AD intervention.

Excessive Aβ aggregation into plaques is widely considered one of the major pathological changes that occur in the brain of AD patients [51]. To date, the removal of pathogenic proteins or abnormal aggregates (Aβ deposition and hyperphosphorylated tau protein accumulation) in the brain is still the primary option for AD treatment [52]. In our work, THz exposure not only remarkably suppressed the formation of Aβ plaques in both the cortex and hippocampus of the AD brain but also significantly reduced Aβ levels, either Aβ42 and Aβ40 or soluble neurotoxic Aβ oligomers, in the brain homogenates (Fig 2), indicating a vital effect of THz on Aβ clearance. Engulfing and degrading Aβ protein *via* microglial activity is the main pathway for Aβ clearance in AD [48]. However, we did not find a significant change in the uptake of Aβ by microglia in the brain of AD mice irradiated by THz compared with the AD control group (Fig S3). We hypothesize that this may be due to the excessive inflammatory response including microglial activation in the AD brain microenvironment. while THz stimulation does not affect the dysfunctional microglia. Previous evidence has suggested that AMPK signaling pathway activation can decrease Aβ production [53]. Furthermore, mTOR-dependent signaling pathway activation can upregulate autophagy activity and facilitate the lysosomal degradation of Aβ [54]. Non-invasive stimulation, such as repetitive transcranial magnetic stimulation for AD treatment, has a potential mechanism related to the activation of β-catenin and the promotion of neuronal survival [55]. The direct action of THz in AD on neural pathways needs further study. Since THz irradiation could disrupt the hydrogen-bond network within the amyloid protein, it can be expected that THz exposure leads to a conformation change of the Aβ peptide. Previously, Kawasaki et al. found that intense THz irradiation dissociates the fibrous conformation of the amyloid peptide by decreasing the β-sheet while increasing the α-helix [56]. Tang et al. reported that Aβ aggregation states display distinct dielectric responses to THz waves [57]. One most recent study on Aβ oligomers *in vitro* has revealed that THz waves of a specific frequency suppress the fibrotic process of Aβ [58]. Further investigation of molecular dynamics has revealed that THz waves resonate with Aβ fibrils and disrupt the dense conformation by breaking the β-sheet structure [58]. This may provide evidence for the molecular mechanism underlying the effects of THz on Aβ aggregation and conformation. This study also laid the groundwork for our animal trials of THz treatment for AD.

Therefore, we presume that THz-induced conformational changes not only inhibit the amyloid fibril formation but form Aβ states that favor physiological clearance. As a consequence, the reduced Aβ level in the brain of AD mice decreased the microglia and astrocytes activation and suppressed neuroinflammation (Fig 6). This is consistent with previous studies showing that neuroinflammation increases Aβ production, and aggregated Aβ triggers microgliosis and astrogliosis, resulting in a proinflammatory state [59, 60].

Overall, the current study demonstrates that low-frequency THz waves can ameliorate the cognitive deficits in APP^SWE^/PS1^DE9^ double transgenic mice of AD by decreasing Aβ/tau pathology, rescuing neuronal loss, and ameliorating neuroinflammation and mitochondrial function. When incorporated into the growing body of evidence that physical treatment might be useful for AD therapy [17–20], our findings provide a basis for THz treatment as a strategy against AD. However, due to the unique physical properties of THz waves, such treatment of AD may have a variety of biological effects on the nervous system, including promoting nerve growth [61], altering membrane permeability [28], and regulating neurotransmitter levels *in vitro* [62].

We also found that THz waves treatment significantly attenuated the loss of NeuN-positive neurons and dendritic density in the hippocampus of 8-month-old male AD mice (Fig 5) and mitigated mitochondrial dysfunction (Fig 7). This may be another potential mechanism by which THz waves rescue the cognitive deficits and related neuropathology in AD. The detailed cellular and molecular mechanisms underlying the beneficial effects of low-frequency THz on AD need to be further investigated in the future.

### Supplementary Information

Below is the link to the electronic supplementary material.

Supplementary file1 (PDF 474 KB)

## Data Availability

All the data and methods needed to evaluate the conclusions of this work are presented in the main text and the Supplementary Material. Additional data can be requested from the corresponding author.
