# Supplementary material for: Terahertz Irradiation Improves Cognitive Impairments and Attenuates Alzheimer’s Neuropathology in the APPSWE/PS1DE9 Mouse: A Novel Therapeutic Intervention for Alzheimer’s Disease
Source: Neurosci Bull. 2023 Nov 16;40(7):857–71. doi: 10.1007/s12264-023-01145-3 (PMC11250709; doi:10.1007/s12264-023-01145-3)
Supplement: Supplementary file 1 — Supplementary file1 (PDF 474 KB) [file 12264_2023_1145_MOESM1_ESM.pdf]

Supplementary Materials

Supplementary Methods

RotaRod Test

The RotaRod test was used to evaluate the motor ability and balance of mice. The experiment was divided into two sessions. The first was the training session, in which the mice were trained for 4 consecutive days, 3 times a day, from the minimum rotation speed to self-falling, with a 1 h rest interval each time. The second session was the test session on day 5, in which the rotation time and maximum rotation speed when the mice fell were recorded.

Statistics

The results are presented as the mean ± SEM. As applicable, statistical comparisons between two groups were made using Student's *t*-test or the Mann–Whitney U-test. One-way ANOVA and Tukey's test were used to compare four groups of mice. P values <0.05 (two-sided) were considered significant. All analyses were performed with GraphPad Prism software, version 9.0.

Supplementary Figures and Figure Legends

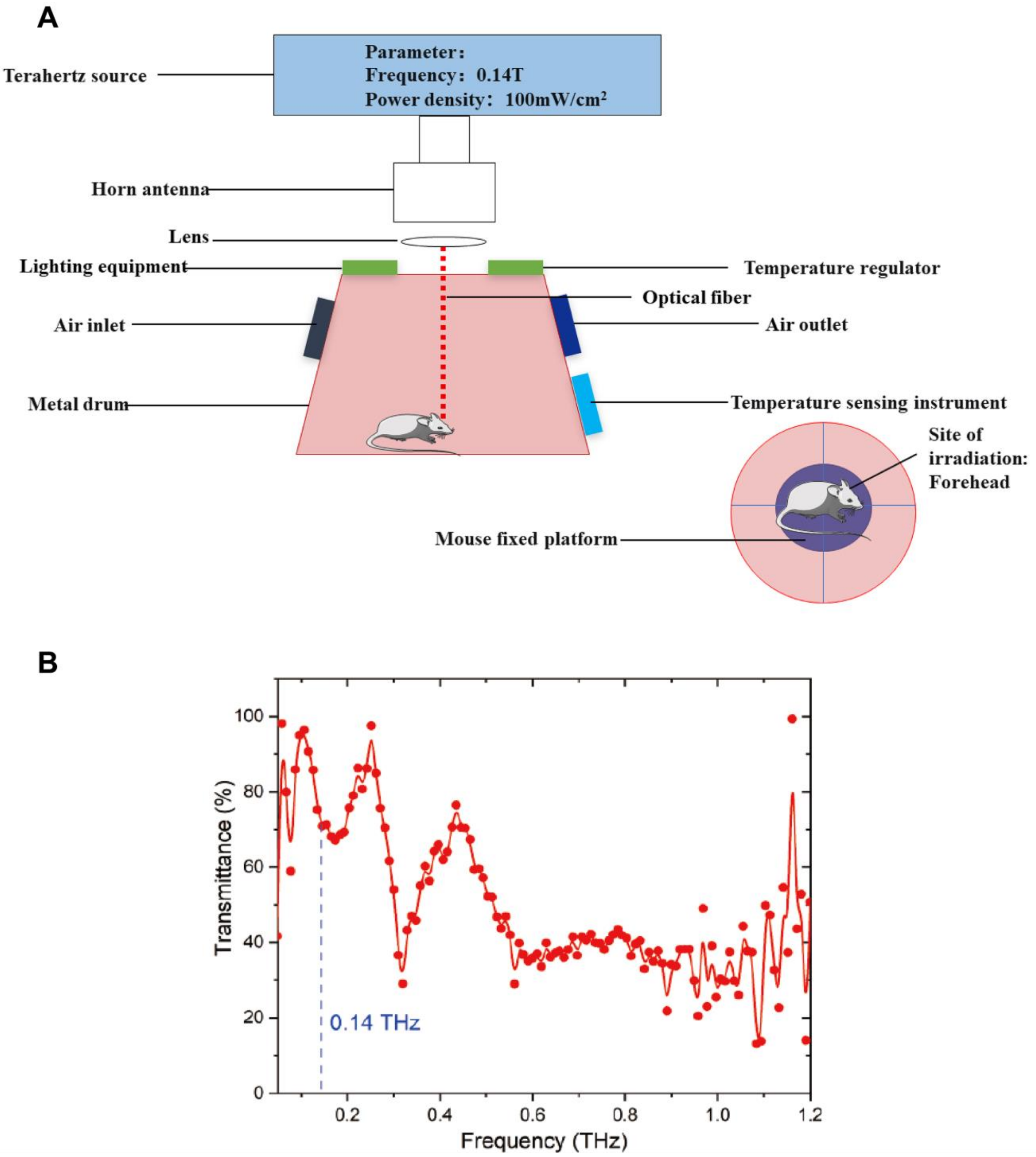

Fig. S1 THz irradiation apparatus and the transmittance of 0.14 THz waves. A The THz waves irradiation system setup: THz waves emitted by the THz source are fed into a horn antenna and collimated using a TPX lens to obtain a beam. The beam is transmitted through an optical fiber, illustrated by the red dotted line, which can transmit the THz waves to the forehead of the experimental animal. The temperature, ventilation, and illumination systems are all in operation to maintain a relatively stable irradiation environment. **B** The transmittance of calvaria was obtained by comparison with the reference signal (air), indicating that the transmissivity at 0.14 THz (the frequency used in this study) exceeds 70%, and THz can penetrate calvaria and act on the relevant brain regions.

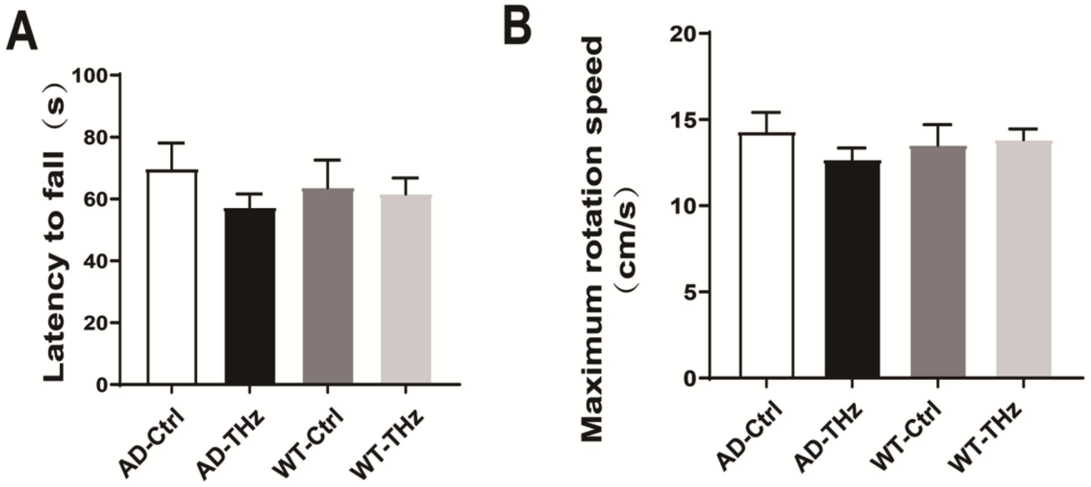

Fig. S2 The motor ability and balance of mice are intact after THz irradiation. A The latency to falling. **B** The maximum rotation speed. *n* = 10 per group. Data are presented as the mean ± SEM. AD: $APP^{SWE}/PS1^{DE9}$ transgenic mice; Ctrl: control; WT: wild type.

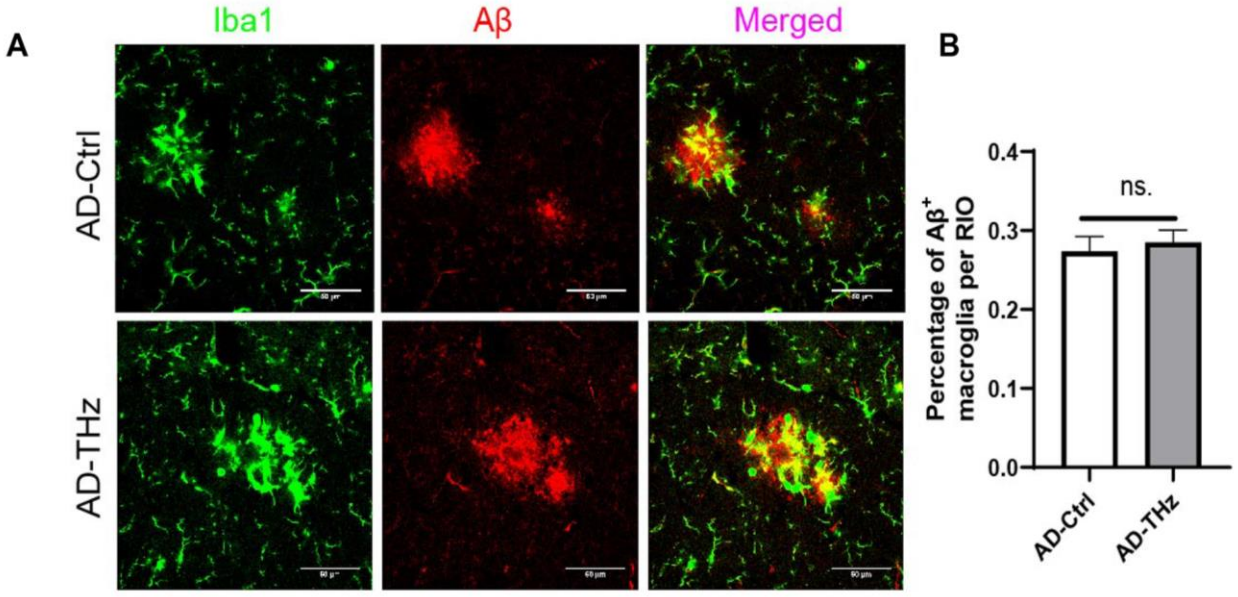

Fig. S3 THz waves have no effect on Aβ uptake by microglia in the brain of AD mice. A Confocal stack images of colocalization of microglia with Aβ plaques in the hippocampal region. **B** Comparison of the immunoreactive area of co-localized Aβ and IBA1 in the brain of $APP^{SWE}/PS1^{DE9}$ mice between the two groups. Scale bars 50 μm. *n* = 10 per group. Data are presented as the mean ± SEM. AD: $APP^{SWE}/PS1^{DE9}$ transgenic mice; Ctrl: control.

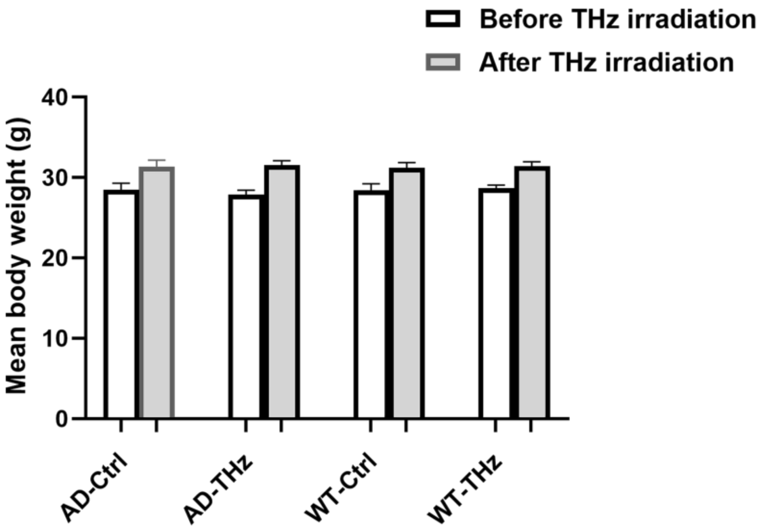

Fig. S4 Body weight of mice before and after THz irradiation in each group. Data are expressed as the mean ± SEM (WT-Ctrl, n = 18; WT-THz, n = 18; AD-Ctrl, n = 18; AD-THz, n = 18). AD: $APP^{SWE}/PS1^{DE9}$ transgenic mice; Ctrl: control; WT: wild type.

Table S1. Antibodies used in this study.

Target	Species	Application	Dilution	Company	Cat. number
6E10	Mouse	IFC	1:800	Covance	SIG-39300
6E10	Mouse	WB	1:1000	Covance	SIG-39300
Iba1	Rabbit	IFC	1:1000	Wako	019-19741
GFAP	Rabbit	IFC	1:2000	Abcam	ab7260
MAP-2	Rabbit	IFC	1:200	Abcam	ab183830
NeuN	Mouse	IFC	1:500	Merck	MAB377
COX-IV	Rabbit	IFC	1:200	Beyotime	AF2047
p-tau S396	Rabbit	IFC	1:1000	Abcam	ab109390
p-tau S396	Rabbit	WB	1:1000	Abcam	ab109390
p-tau 231	Rabbit	IFC	1:200	Abcam	ab151559
p-tau 231	Rabbit	WB	1:1000	Abcam	ab151559
Tau5	Mouse	WB	1:1000	Abcam	ab80579
Synaptophysin	Rabbit	IFC	1:1000	Abcam	ab32127
GAPDH	Rabbit	WB	1:4000	CST	2118
